# Basal cell carcinoma with compromised margins: retrospective study of management, evolution, and prognosis^☆^^☆☆^

**DOI:** 10.1016/j.abd.2020.11.001

**Published:** 2020-11-20

**Authors:** Maria Carolina Fidelis, Rafael Fantelli Stelini, Leonardo Piropo Staffa, Aparecida Machado de Moraes, Renata Ferreira Magalhães

**Affiliations:** a Department of Dermatology, Hospital das Clínicas, Universidade Estadual de Campinas, Campinas, SP, Brazil; b Department of Pathological Anatomy, Hospital das Clínicas, Universidade Estadual de Campinas, Campinas, SP, Brazil; c Ophthalmology Service, Hospital das Clínicas, Universidade Estadual de Campinas, Campinas, SP, Brazil; d Faculty of Medicine, Universidade Estadual de Campinas, Campinas, SP, Brazil

**Keywords:** Follow-up studies, Carcinoma, basal cell, Neoplasm recurrence, local

## Abstract

**Background:**

Non-melanoma skin cancer is the most common type of malignancy in the Western world, and surgical excision is the preferred approach. The approach adopted in the face of incomplete excisions of basal cell carcinoma is still controversial.

**Objectives:**

To compare the number of tumor recurrences after treatment for incompletely excised basal cell carcinoma.

**Methods:**

Selection and statistical analysis of medical records of patients who had compromised margins after excision of basal cell carcinoma in a tertiary hospital from 2008 to 2013.

**Results:**

A total of 120 medical records were analyzed; the mean age was 69.6 years, and 50% of the patients were female. The most prevalent histological type was nodular; the mean size was 1.1 cm, and the tumor location with the highest incidence was the nose. The lateral margin was the most frequently positive. Clinical follow-up was more widely adopted; only 40 patients underwent a second surgery. The total number of patients who had tumor recurrence was 34 (28.3%). Only the malar location significantly influenced the incidence of recurrence (p = 0.02). The mean follow-up time was 29.54 months, with no significant difference between the follow-ups, although 32.9% of the patients followed-up clinically showed recurrence, against only 20% of those who underwent a second surgery.

**Study limitations:**

Mean follow-up time of less than five years and sample size.

**Conclusions:**

The presence of compromised margins does not necessarily imply recurrence. Location, tumor size, histological subtype, previous epithelial tumors, and clinical conditions of the patient must be considered when choosing the best treatment option.

## Introduction

Skin cancer is the most common malignancy in the Western population; in Brazil, it accounts for approximately 30% of all reported malignant tumors.^1^, ^2^, ^3^ Data for non-melanoma skin cancer (NMSC) provided by the National Cancer Institute (Instituto Nacional de Câncer [INCA]) estimate an incidence of 176,930 new cases in 2020, 83,770 in men and 93,160 in women, and a mortality rate of 1.1%.^2^ Basal cell carcinoma (BCC) accounts for approximately 75% of NMSC, followed by squamous cell carcinoma (SCC).^4^, ^5^, ^6^ BCC is a malignant tumor of follicular germ cells; it mainly affects individuals from the sixth decade of life onwards, with a history of chronic sun exposure, and is located more often in the head and neck, followed by the trunk, limbs, and genitals.^4^, ^7^, ^8^ It presents slow growth, which can lead to tissue destruction due to its locally aggressive behavior, and low metastatic potential, from 0.0028% to 0.55%.^9^, ^10^ Although its mortality is low, it has high morbidity, being one of the most expensive of all cancer treatments evaluated in the Medicare system in the United States.^11^

Ultraviolet (UV) radiation is regarded as the major cause of BCC.^12^ The risk of developing BCC is increased by recreational sun exposure during childhood and adolescence, in addition to exposure to intermittent and intense UV radiation (sunburn).^13^ Other risk factors include male gender, Fitzpatrick type I and II, personal history of BCC, chronic exposure to arsenic, exposure to ionizing radiation, long-term immunosuppression, genetic syndromes (BCC syndrome, xeroderma pigmentosum), and family history. Scars and chronic ulcerations are important for the onset of BCCs in areas not exposed to UV rays.^14^

The diagnosis of BCC is made by clinical examination, dermoscopy, and histopathological examination.^15^ Clinical examination demonstrates the previously described characteristics, such as a pearly appearance and the presence of telangiectasias, together with slow growth, especially in places exposed to the sun in fair-skinned individuals. Dermoscopy allows the identification of structures described for BCC, such as arborizing telangiectasia, thin and short telangiectasias, spoke-wheel-like structures and leaf-like structures, in addition to blue-gray globules and ovoid nests.^16^, ^17^ Histopathological examination is essential to confirm diagnosis and estimate the risk of recurrence.^18^

The nomenclature of the morphological presentations of the BCC varies according to each author; the following subtypes can be distinguished based on their different clinical and histological appearance: nodular BCC, superficial BCC, and sclerodermiform BCC.^19^ The nodular subtype is responsible for 50% to 80% of all cases of BCC.^20^ The micronodular, infiltrative, sclerodermiform, and basosquamous histological subtypes are associated with more aggressive behavior, such as higher rates of recurrence and greater invasion of the dermis.^21^

Treatment options for BCC include conventional surgical excision, Mohs micrographic surgery, curettage with or without electrodissection, cryosurgery, photodynamic therapy, radiotherapy, and drug therapy with topical, oral, or intralesional injections.^15^, ^22^ Treatment options are dictated by the size of the tumor, location, histological subtype, treatment adherence, medical comorbidities, and cosmetic result.^22^

Conventional surgical excision is the treatment of choice for most BCCs, being the most commonly adopted. Although there are several treatments for low-risk BCC, such as topical therapy with imiquimod, surgery is considered the gold standard.^18^, ^23^ In order to decrease the number of incomplete excisions, a peritumoral margin of at least 4 mm of clinically normal skin is the standard for conventional surgical excision of BCCs.^22^, ^23^ For noble anatomical structures, such as areas of the face, this margin can be considered large; it can be reduced to less than 4 mm and assessed during the intraoperative period using the Mohs technique.^18^ High-risk BCC is associated with a higher incidence of local recurrence, subclinical tumor extension, incomplete excision, and aggressive behavior.^17^

For patients who had incomplete BCC excisions, some authors opt for re-excision, while others opt for clinical follow-up. Approaching a tumor with compromised margins is challenging. Thus, studies that evaluate the evolution of the management and the prognosis are extremely important, since they can reduce morbidity.

The present study aimed to analyze the cases of BCCs that were operated on in a tertiary hospital between 2008 and 2013 and whose margins were compromised, seeking to evaluate the type of conduct adopted (whether clinical follow-up or re-excision) in order to compare the results obtained, as well as all the factors that could possibly be associated with these results.

## Methods

### Sample characterization and data collection

The medical records of patients who had incomplete excised BCCs from 2008 to 2013 using the PathoControl*®* software were separated for analysis. The keywords for search, in addition to “basal cell”, were: “small”, “slender”, “narrow”, “leaning”, and “compromised”, both in singular and plural, as well as in uppercase and lowercase letters.

The medical records of patients who died, were under the age of 18, were operated on by specialists other than dermatologists, or those that presented incomplete data were excluded. The medical records of patients who underwent only biopsy, partial excision, minimum follow-up of less than three months, or who obtained tumor-free margins were also excluded; thus, a total of 120 medical records were analyzed.

The variables studied included: age, sex, location, histological subtype, tumor size, type of compromised margin (lateral, deep, and both), type of follow-up (clinical or surgical), type of surgical approach (conventional or Mohs), presence or absence of residual tumor in the re-excision, recurrence, and time of follow-up.

This study was approved by the Research Ethics Committee. Opinion number: 3,515,060.

### Analysis of medical records

The variables were analyzed descriptively, comparing absolute and percentage frequencies and, statistically, comparing the relationship between these variables and tumor recurrence. A third analysis took into account the patients' follow-up time, allowing the establishment of disease-free time relationships.

The descriptive analysis was performed with frequency tables for categorical variables, and measures of position and dispersion for numerical variables. To compare numerical variables between the two groups, the Mann-Whitney test was used; to compare proportions, the chi-squared test or Fisher's exact test were used.

In the statistical analysis, p-values were obtained using Fisher's exact test (for groups with n ≤ 5) and the chi-squared test, both two-tailed. The recurrence-free time was analyzed using Kaplan-Meier survival curves and the groups were compared using the log-rank test. All analyses considered the level of statistical significance within a confidence interval of p < 0.05. The SAS System for Windows (Statistical Analysis System – v. 9.4. SAS Institute Inc., 2002–2008, Cary, NC, United States) was used for statistical analysis.

## Results

The study included 120 patients, with a mean age of 69.6 years and a median of 71.5 years, ranging from 31 to 100 years. There were 60 male patients (50%) and 60 female patients (50%).

**Figure 1:**
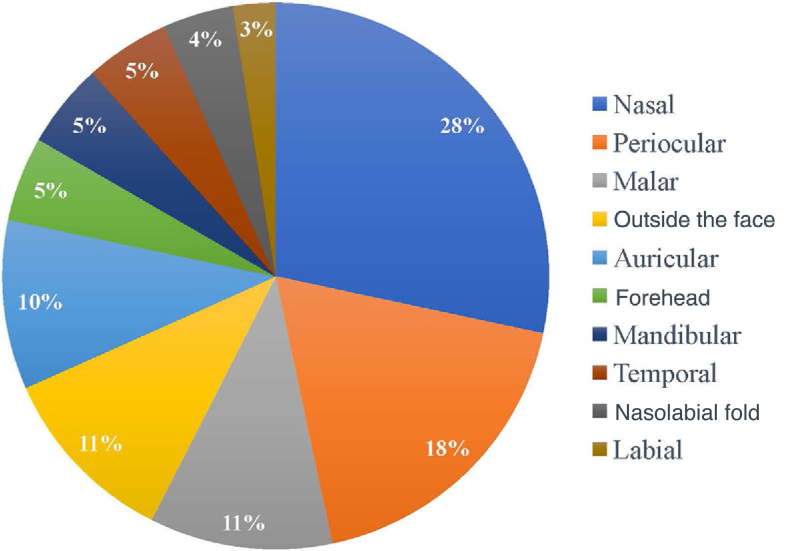
Tumor site.

Regarding the location of the lesions, the highest incidence of tumor was in the nasal area, in 34 patients (28.33%), followed by periocular (22 patients; 18.3%) and malar (13 patients; 10.8%) regions. Only 13 of the 120 patients (10.8%) had a tumor outside the face, as shown in Fig. 1. As for the ethnicity of the patients analyzed, only one patient was mixed-race (0.8%), while 119 were white (99.2%).

**Figure 2:**
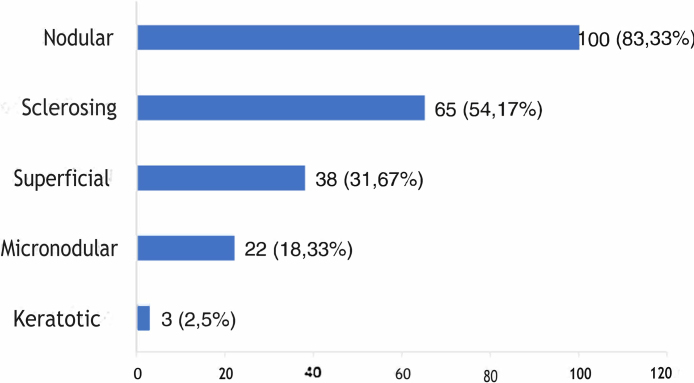
Histopathological subtypes of the tumors.

The histological types found overlapped, with the same tumor showing different histological patterns, totaling 228 occurrences. These were grouped into nodular, superficial, sclerosing, micronodular, and keratotic. The four main patterns found were: nodular (83.33% of tumors), sclerosing (54.17%), superficial (31.67%), and micronodular (18.33%), as shown in Fig. 2, in absolute values and percentages.

The mean tumor size was 1.1 cm and the median was 0.8 cm, the smallest measuring 0.3 cm and the largest, 5.2 cm. The charts of only 50 patients presented a description of tumor size.

**Figure 3:**
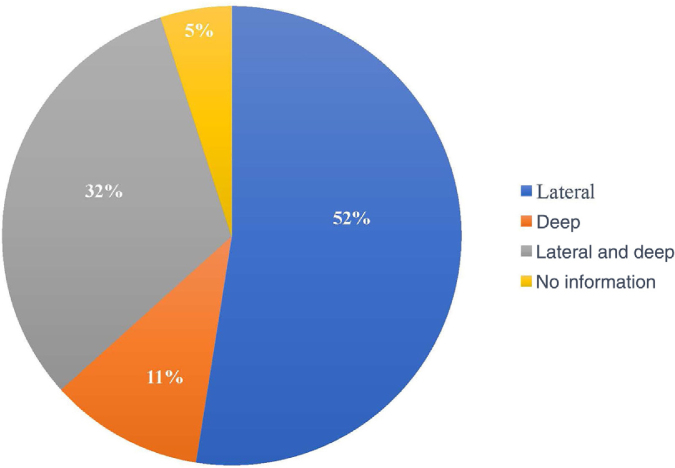
Compromised margins.

The compromised margin with the highest incidence was the lateral margin, in 63 excisions (52.5%); the deep margin was compromised in 13 (10.8%); 38 patients presented both the deep and lateral margins compromised (31.7%). Of the 120 anatomopathological reports, 114 specified the compromised margin, as shown in Fig. 3.

**Figure 4:**
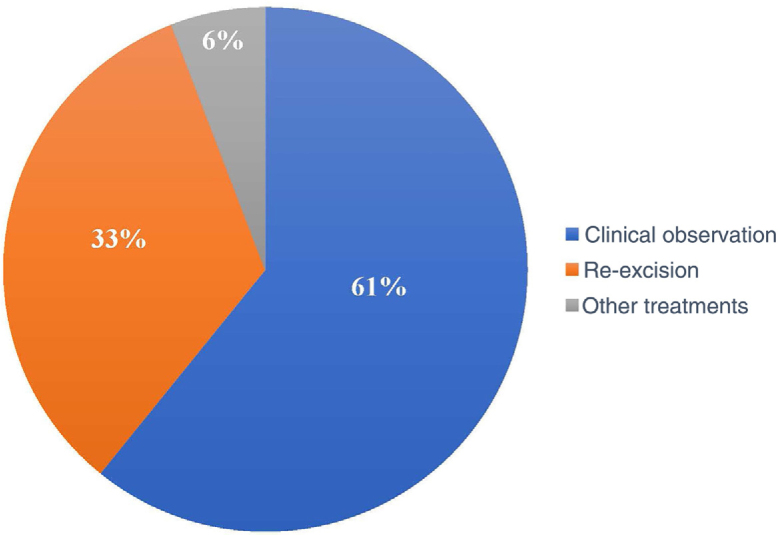
Conduct adopted. The term “other treatments” refers to patients who underwent non-surgical treatments.

Of the 120 patients, 73 (60.8%) underwent clinical follow-up of the compromised margins, while 40 (33.3%) underwent re-excision. The remaining seven underwent non-surgical treatments: four underwent radiotherapy, two underwent cryosurgery, and one was treated with topical imiquimod (Fig. 4).

Of the 40 patients who underwent re-excision, conventional surgery was performed in 30 (75%), while ten (25%) underwent Mohs micrographic surgery. Among these 40 patients, 23 (57.5%) presented residual tumor *vs*. 17 (42.5%) without residual tumor.

Frequencies of recurrence were compared, considering the following as independent variables: age, sex, location, tumor size, type of affected margin, type of follow-up (observation or re-excision), type of re-excision (conventional or Mohs), and presence or absence of residual tumor. The frequencies for each histological pattern were not compared due to the overlap of different subtypes in the same tumor. The association between tumor factors is shown in Table 1.

**Table 1:** Association of selected factors with tumor recurrence.

	Recurrence		Total	% Recurrence	p
	Yes	No			
**Age (years)**					
Mean ± SD	67.6 ± 13.6	70.4 ± 13.6	69.6 ± 13.6	N/A	0.4540^a^
Median (min–max)	71.5 (31.0–85.0)	71.5 (37.0–100.0)	71.5 (31.9–100.0)	N/A	
Total	34	86	120		
**Sex**					
Males	15	45	60	25	0.4178^b^
Females	19	41	60	31.7	
Total			120		
**Location**					
Nasal	11	23	34	34.3	0.5389^b^
Periocular	6	16	22	27.3	0.9028^b^
Malar	0	13	13	0	0.0186^c^, ^d^
Auricular and periauricular	2	10	12	16.7	0.7264^c^
Forehead	4	2	6	66.67	0.0533^c^
Mandibular	3	3	6	50	0.3497^c^
Temporal	1	4	5	20	0.5139^c^
Nasolabial groove	2	4	6	33.3	0.5211^c^
Lip	1	2	3	33.3	1.0000^c^
Outside the face	4	9	13	30.8	0.8562^c^
Total	34	86	120		
**Size (cm)**					
Mean ± SD	1.5 ± 1.4	1.0 ± 0.6	1.1 ± 0.9	N/A	0.1932^a^
Median (min–max)	0.9 (0,3–5,2)	0.8 (0,3–2,5)	0.8 (0,3–5,2)	N/A	
Total	14	36	50		
**Compromised margin**					
Lateral	18	45	63	28.6	0.4453^b^
Deep	5	8	13	38.5	
Lateral and deep	8	30	38	21	
No information	3	3	6	50	
Total			120		
**Type of follow-up**					
Clinical observation	24	49	73	32.9	0.3481^b^
Re-excision	8	32	40	20	
Other treatments	2	5	7	28.6	
Total			120		
**Type of re-excision**					
Conventional	6	24	30	20	1.0000^c^
Mohs	2	8	10	20	
Total			40		
**Residual tumor**					
Present	5	18	23	21.7	1.0000^c^
Absent	3	14	17	17.6	
Total			40		
**Total**	34	86	120	28.3	N/A

N/A, Not applicable.

The p-values were obtained:

a Based on the Mann-Whitney test;

b Based on the chi-squared test;

c Based on Fisher's exact test.

d p < 0.05 using Fisher's exact test.

The mean age of patients who did not present recurrence was 70.4 years and the median was 71.5 years (ranging from 37 to 100), whereas the mean age of those who presented recurrence was 67.6 years and the median, 71.5 years (ranging from 31 to 85). No statistically significant difference was observed between the groups.

A higher number of recurrences (55.9%) was observed in the female patients when compared with the males (44.1%), but the difference was not statistically significant. The mixed-race patient did not present recurrence, and it was therefore not possible to calculate the p-value.

Of the total of 120 cases of incomplete BCC excision, 86 did not show recurrence, *vs.* 34 cases of recurrence. Regarding the location of the tumor, the malar region did not present any cases of recurrence, a statistically significant difference (p < 0.05); in turn, the difference for other regions was not statistically significant. Ten cases in the auricular or periauricular region did not present recurrence (11.6%), which was observed in two cases (5.9%). A total of 16 cases in the periocular region did not show recurrence (18.6%), which was observed in six cases (17.6%). Two cases in the forehead did not show recurrence (2.3%), which was observed in four cases (11.8%). The mandibular region presented three cases (8.8%) of recurrence and three cases with no recurrence (3.5%). The temporal region presented only one case of recurrence (2.9%), which was not observed in the remaining five cases (5.8%). Three (3.5%) cases in the nasolabial groove did not show recurrence, observed in two cases (5.9%). Of the three cases in the labial region, two did not recur (2.3%), while one did (2.9%). Outside the face, there were four cases (11.7%) of recurrences and nine (10.5%) did not recur; the nasal region presented the highest number of recurrences, 11 (32.4%), while 23 cases (26.7%) did not present recurrences.

There are studies claiming that BCC aggressiveness is related to larger lesion diameters and subclinical extension, so the present authors decided to analyse the scarce data on mean lesion size (n = 50). Among patients who did not have recurrence, the mean lesion size was 1.0 cm and the median was 0.8 cm (0.3–2.5), while in those who had recurrence the mean size was 1.5 cm and the median was 0.9 cm (0.3–5.2); however, this difference was not statistically significant.

No difference was found in recurrence regarding the type of affected margin: lateral, deep, or both. Regarding the type of follow-up, despite the apparently higher proportion of recurrences in patients who underwent clinical follow-up when compared with those who underwent a second surgery (32.9% *vs.* 20% respectively), the difference between the two approaches was not statistically significant. There was also no difference between the types of second surgery (conventional or Mohs) or the presence or absence of residual tumor.

**Figure 5:**
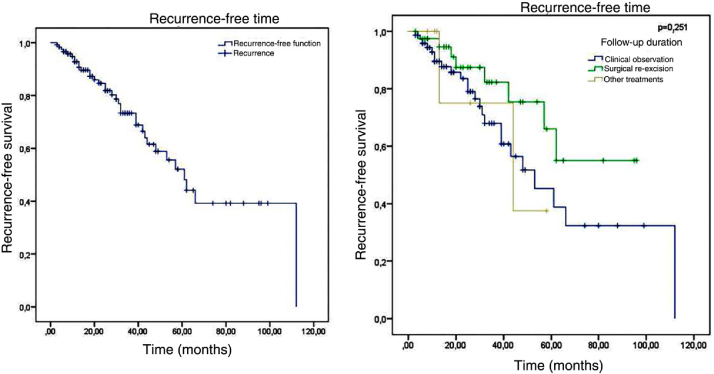
Kaplan-Meier survival analysis for recurrence-free time and recurrence-free time according to the type of follow-up.

Of the 120 patients, 34 presented recurrence and 86 remained tumor-free, which indicates a final tumor recurrence rate of 28.3%. The mean follow-up time was 29.54 months, with a standard deviation of 23.07 months. The median was 25 months, ranging from 3 to 112 months, which indicates that half of the patients were followed-up for over two years. No statistically significant difference (p = 0.251) was observed in the recurrence-free time in patients who were clinically followed-up, those who underwent re-excision, and those who underwent other treatments. The Kaplan-Meier survival analysis for recurrence is shown in Fig. 5.

**Figure 6:**
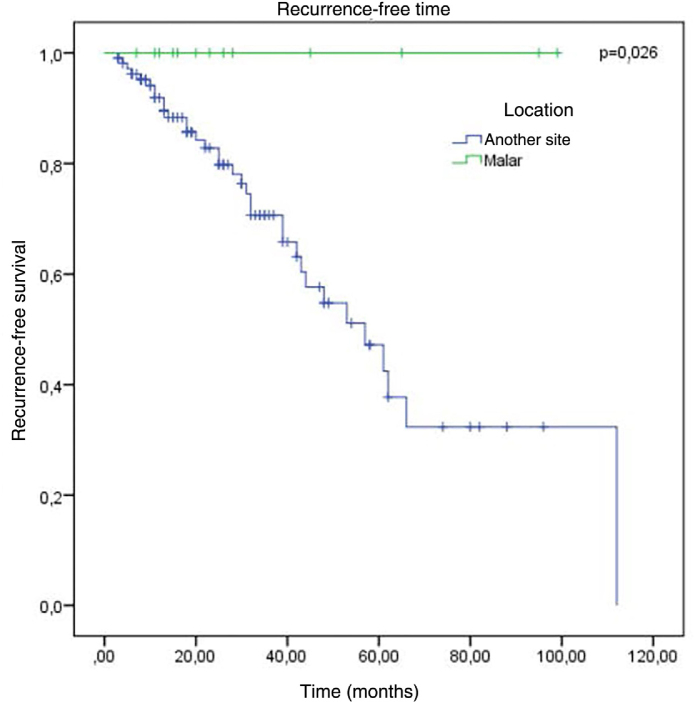
Kaplan-Meier survival analysis for recurrence according to location.

The factors studied were related to the time that each patient remained free of tumor recurrence, and no statistically significant difference was observed in disease-free time in relation to any of the factors studied, except for patients with tumor in the malar region (p = 0.026; Fig. 6).

## Discussion

The mean age of the patients with a compromised margin was compatible with the findings of other studies.^18^, ^24^ The data demonstrate that, in general, the lesions appear initially after the sixth decade of life, despite the occurrence of cases diagnosed before the age of 30. However, cases with a compromised margin tend to have a higher mean age, ranging from 66 to 74 years, according to the findings in the literature.^18^, ^21^, ^25^, ^26^, ^27^, ^28^ Studies observed a statistically significant difference between the mean age of the group of patients who had complete excision and the group of patients with incomplete excision; the second group had a higher age.^25^, ^28^

In the present study, no statistically significant difference was observed in the age of presentation between men and women, and incomplete excisions did not occur preferentially in a given sex. However, another study pointed out that women may have more compromised margins than men due to undergoing more conservative surgeries, for better esthetic results.^29^

Most tumors observed in this study were less than 2 cm, denoting patients' demand for care and a correct and early diagnosis, as observed in other studies.^25^, ^27^ Despite the controversial influence of tumor size on recurrence rates, data from the literature demonstrate that there is a wide variation in subclinical extent in terms of tumor diameter.^22^, ^23^ Another important aspect that can be defined based on the size of the lesion is the surgical margin. Studies indicate that a margin of 4 mm is adequate to eradicate primary BCC lesions smaller than 2 cm in diameter.^30^

The nasal region is the one with the most frequently compromised margins (28%), followed by the periocular region (18%). Authors cite a rate of positive margins ranging from 14% to 40% in the nasal region and from 11% to 85% in the periocular region.^18^, ^21^, ^27^, ^30^^,^^31^ The reason for the greater number of incompletely excised BCCs in these regions may be the result of inadequate margins for better esthetic results in these locations.^25^

Regarding histopathology, there was an overlap of different histological subtypes: the nodular type had the highest incidence (83.33%), followed by sclerosing (54.17%), superficial (31.67%), micronodular (18.33%), and keratotic (2.5%). This finding is consistent with the literature, in which nodular BCC is the most commonly reported clinical variant, with an incidence greater than 85%.^18^, ^32^, ^33^, ^34^, ^35^ No statistically significant difference was observed between the histopathological subtype and recurrence, although most references emphasize that tumors with a more aggressive histological subtype are associated with a higher incidence of recurrence.^36^

In most studies, the lateral margin was the most compromised, similar to what was in found the present study.^18^, ^21^, ^27^, ^30^^,^^31^ This could be explained by the subclinical extent of the lesions, surgeries in areas with anatomical restrictions, and by the surgeon's natural tendency to preserve tissue more laterally than deeply.^21^, ^31^, ^37^

In the period considered by this study, the preferred approach was clinical follow-up (60.8%) instead re-excision (33.3%) and other treatments (5.8%). Some authors advocate re-excision in most cases with a positive margin, while others suggest re-excision for only recurrent cases and aggressive subtypes.^27^, ^37^ Another group of authors advocates a conservative approach, especially in patients with multiple comorbidities.^28^

The adoption of an expectant follow-up may be acceptable in some cases of positive surgical margins, such as after excision of BCC in low-risk locations (trunk, extremities, scalp and malar region), for histopathological subtypes of less aggressiveness, and in patients with advanced age or comorbidities.^28^, ^30^, ^38^ However, incomplete BCC excision, especially on the face, can result in greater morbidity if the follow-up is observational, as recurrence may require a more extensive surgical procedure.^39^ It must also be considered that incomplete excision implies an increase in the demand for specialized care services and in the costs of a second procedure, which is generally more complex.^25^, ^26^, ^40^

In patients in whom re-excision was the adopted follow-up, the presence of residual tumor was observed in 57.5% of re-excisions. Such incidence is consistent with the literature, comparable to that obtained by Masud et al.,^31^ of 62.9%.

The total recurrence found after incomplete tumor excision was 28.3%. Most studies reported higher values, ranging from 38% to 46%.^21^, ^41^, ^42^, ^43^ However, other authors observed a recurrence rate of 25%, attributing this occurrence to a shorter follow-up time than other studies.^44^ One study found that 82% of the recurrences occurred in the first five years, and only about 30% of cases were diagnosed in the first year of follow-up.^38^ It is also estimated that excision of BCC without margin control may result in a five-year recurrence rate of 3.2%–10.0% for primary BCC and 12.1%–17% for recurrent BCC.^37^, ^43^, ^44^, ^45^

Then, a limitation of the present study may be the duration of the follow-up period (median of two years). The recommended follow-up for recurrence is five years; consultations should be held every three months during the first year and then every six months.^46^

The mean age among the 34 patients who presented tumor recurrence was 67.6 years, while that of the 86 patients who did not show recurrence was 70.4 years; this difference was not statistically significant. Furthermore, no difference was observed in age between the three groups, in contrast to the findings in the literature, which found a higher mean age in the group of patients who underwent re-excision.^25^ Factors such as sex and variables inherent to the tumor or to the surgical technique were not significantly associated with greater tumor recurrence.

In order to decrease the number of incomplete excisions, a peritumoral margin of at least 4 mm of clinically normal skin is the standard for conventional surgical excision of BCCs.^39^, ^41^ However, cure rates may decline in cases of larger tumors (> 2 cm), aggressive histological subtypes, and tumors in specific locations.^13^ In the present study, almost all recurrences (approximately 83%) occurred in cosmetically noble areas, where the peripheral margin may have been smaller.

Regarding tumor location, most BCCs with compromised margins are located on the head and neck; the nasal and periocular regions are the places where recurrent tumors are most frequently located, followed by the periauricular region.^18^, ^25^, ^37^ In agreement with the literature, it was observed that the highest rate of recurrence was in the nasal (32.4%), periocular (17.6%), and periauricular (11.6%) regions. A published meta-analysis on this subject found a 2.24 higher relative risk of positive margins for BCCs in the nasal and periauricular region than in the rest of the body.^47^ This occurrence could be justified by the fact that these regions are cosmetically noble, which would hinder the choice of the appropriate margin.^3^

No association was observed with the regions with the highest incidence of recurrence. The only factor that showed a significant association with a lower chance of recurrence was the malar location, where there were no cases of recurrence (p < 0.05).

Regarding the management in relation to the compromised margins, the main objective of this study, a total recurrence rate of 32.9% was found in patients with clinical follow-up, 28.6% of those who underwent other treatments, and 20% in those who underwent re-excision, which could suggest greater success of the surgical approach over the other approaches when disregarding the follow-up time; however, the p-value of 0.14 indicates that the difference was not statistically significant.

Large tumors (greater than 0.8 cm) and only deep margins (38.5%) presented more cases of recurrence, but without statistical significance. A pioneering study by Wetzel et al.^36^ indicates that BCCs of any subtype with aggressive histological characteristics have a greater depth of invasion, statistically significant, when compared with non-aggressive BCCs, a factor that may justify the incidence of recurrence in patients with deep compromised margins. According to these authors, the median depth of invasion of all BCCs, regardless of other histopathological characteristics and subtype, was 0.79 mm, ranging from 0.10 to 5.49 mm.

These findings may explain the high rate of recurrence when the type of follow-up adopted was “other treatments.” In any topical therapy, the adequate penetration of a drug to its target can be a concern with agents such as imiquimod; similar concern can be raised for determining the prescribed dose of radiotherapy.^48^

Higher proportions of recurrences were also observed for conventional surgery when compared with the Mohs technique. All of these characteristics that potentially indicate a higher risk were consistent with other studies on CBC recurrence.^21^, ^41^, ^42^, ^43^ However, this higher risk is considered only potential because, while many of these characteristics were shown in other studies as being associated with higher rates of recurrence, in the present study the statistical analyses failed to reach statistical significance; therefore, the higher risk cannot be considered proven.

These results considered the surgeon's medical specialty: only patients whose BCCs were excised by dermatologists were included. This differentiation is quite common in other clinical studies in which surgical intervention is considered. Ramdas et al.^40^ concluded that BCCs were more often excised with free margins by dermatologists (93%) than by plastic surgeons (83%) and general surgeons (70%).

If the type of follow-up chosen for the patient with a positive margin is clinical observation, it is necessary to educate the patient about the possibilities of active prophylaxis against BCC, especially in relation to sun protection. Furthermore, it is also necessary to inform the patient about the need for self-examination of the skin. This is of particular importance not only for monitoring recurrence, but also for the risk of developing another BCC, which is ten times higher in patients with previous BCC than in the general population.^45^ This risk is also significantly higher in elderly patients, in patients with multiple BCCs, and those with lesions with a diameter greater than 1 cm.^49^

This study, in line with global algorythms, reiterates the quality of care and treatment offered to patients with BCC in a tertiary hospital.

## Conclusions

The presence of positive margins in the histopathological examination of the tumor does not necessarily imply recurrence. However, an individualized approach is recommended in such cases. Several factors must be considered – particularly tumor location, presence of multiple tumors, and histopathological characteristics – before selecting the best treatment option.

Regarding the conduct adopted in relation to compromised margins, 32.9% of recurrence was observed in patients who were clinically followed-up, *vs.* 20% in those who underwent re-excision. Considering the anatomical location, the present study showed a significant association with a lesser chance of recurrence and a longer time until recurrence in the malar region.

The present data offer new elements for the proper management of patients who present a compromised margin after surgical excision.

## Financial support

None declared.

## Authors’ contributions

Maria Carolina Fidelis: Drafting and editing of the manuscript.

Rafael Fantelli Stelini: Collection, analysis, and interpretation of data.

Leonardo Piropo Staffa: Collection, analysis, and interpretation of data.

Aparecida Machado de Moraes: Intellectual participation in propaedeutic and/or therapeutic conduct of studied cases.

Renata Ferreira Magalhães: Effective participation in research orientation.

## Conflicts of interest

None declared.
